# Changes of the acute myocardial infarction-related resident deaths in a transitioning region: a real-world study involving 3.17 million people

**DOI:** 10.3389/fpubh.2023.1096348

**Published:** 2023-08-21

**Authors:** Yajun Zhao, Jian Zou, Yichen Chen, Jing Zhou, Wei Dai, Minghui Peng, Xiaopan Li, Sunfang Jiang

**Affiliations:** ^1^Department of General Practice, Zhongshan Hospital, Fudan University, Shanghai, China; ^2^Department of Health Management Centre, Zhongshan Hospital, Fudan University, Shanghai, China; ^3^Office of Scientific Research and Information Management, Centres for Disease Control and Prevention, Shanghai, China; ^4^Office of Scientific Research and Information Management, Pudong Institute of Preventive Medicine, Shanghai, China; ^5^School of Public Health, Fudan University, Shanghai, China

**Keywords:** acute myocardial infarction, mortality, years of life lost, comorbidities, trend analysis

## Abstract

**Background:**

The impact of acute myocardial infarction (AMI) on the life span of residents in a transitioning region has not been studied in depth. Therefore, we aimed to evaluate the changes in AMI-related resident deaths in a transitioning region in China.

**Methods:**

A longitudinal, population-based study was performed to analyze the deaths with/of AMI in Pudong New Area (PNA), Shanghai from 2005 to 2021. The average annual percentage change (AAPC) of AMI in crude mortality rates (CMR), age-standardized mortality rates worldwide (ASMRW), and rates of years of life lost (YLLr) were calculated by the joinpoint regression. The impact of demographic and non-demographic factors on the mortality of residents who died with/of AMI was quantitatively analyzed by the decomposition method.

**Results:**

In 7,353 residents who died with AMI, 91.74% (6,746) of them were died of AMI from 2005 to 2021. In this period, the CMR and ASMRW of residents died with/of AMI were 15.23/10^5^ and 5.17/10^5^ person-years, the AAPC of CMR was 0.01% (95% CI: −0.71,0.72, *p* = 0.989) and 0.06% (95% CI: −0.71,0.84, *p* = 0.868), and the ASMRW decreased by 2.83% (95% CI: −3.66,−2.00, *p* < 0.001) and 2.76% (95% CI: −3.56,-1.95, *p* < 0.001), respectively. The CMR of people died of AMI showed a downward trend (all *p* < 0.05) in people ≥60 years but an upward trend [AAPC = 2.47% (95% CI: 0.07,4.94, *p* = 0.045)] in people of 45–59 years. The change in CMR of people died with/of AMI caused by demographic factors was 28.70% (95% CI: 12.99,46.60, *p* = 0.001) and 28.07% (95% CI: 12.71,45.52, *p* = 0.001) per year, respectively.

**Conclusion:**

Preventative strategies for AMI should be applied to enhance the health management of residents aged 45–59 years or with comorbidities in the transitioning region.

## Introduction

Cardiovascular diseases (CVDs) have become one of the most important causes of death in the global population (1, 2). Among the multiple categories of CVDs, acute myocardial infarction (AMI) is a severe and acute clinical syndrome associated with multiple life-threatening comorbidities such as malignant arrhythmia, cardiac shock, and heart failure (3). Post-AMI patients suffer from impaired quality of life, despite the increased use of revascularization therapy such as percutaneous coronary intervention (PCI) to save the ischemic myocardium in current clinical practice (4).

On the other hand, data regarding the long-term trend of the influence of AMI on the resident mortality are relatively lacking in Asia (5), particularly in a transitioning region where there is economic growth, advanced medical services, and aging population. Pudong New Area (PNA) is the largest municipal district of Shanghai, the forefront of China’s reform and opening up, and a typical transitioning area in China (6). The PNA residents’ working environment and living style has drastically changed in recent decades (7).

In this study, by collecting AMI-related mortality data in the death-cause monitoring database of the registered population in PNA from 2005 to 2021, we estimated the trend of the average annual percentage change (AAPC) of AMI in crude mortality rates (CMR), age-standardized mortality rates worldwide (ASMRW), and rates of years of life lost (YLLr) in this population. These real-world data provided us with insight on the influence of AMI on the mortality of the population in this particular region, which may be important for the designating of effective strategies for the prevention of AMI-related deaths.

## Methods

### Data source

Registered residents of PNA were included in the research population, and the data on AMI-related death were collected from 1 January 2005 to 31 December 2021 by screening the death-cause monitoring database of PNA residents. The main reasons we chose the population of PNA as the research object were: (1) The PNA covers an area of 1210.41 km^2^, including both the central urban area and the suburbs (8). The registered resident population is 3.17 million in 2021, accounting for more than 50% of the total population of Shanghai (9). (2) The economy of PNA has experienced a period of rapid development. The total economic volume has increased from 210.8 billion RMB in 2005 to 1,535.3 billion RMB in 2021 (https://www.pudong.gov.cn/006001/20220221/665255.html, First line of the first paragraph), with an average annual growth rate of 13.21%. (3) There are 760,250 people over the age of 65 among the registered residents in 2021, accounting for 23.99% of the entire registered population of PNA (https://wsjkw.sh.gov.cn/cmsres/59/5983e677030440f18a034ce02a0c10e5/d55b3444b8c63b77d3b73231f4f66a28.pdf, in page 9). The huge aging population base and the features of super-aging can provide a more realistic reflection for analyzing the impact of aging on the characteristics of dying with/of AMI. (4) Since 2005, PNA has established a unified, standardized death information registration system based on the whole population, which provides reliable data guarantee for the analysis of AMI death cause (7). Therefore, PNA would be an epitome for most transitioning countries or regions with aging population.

The demographic data in this study came from the PNA Public Security Bureau (PSB). They mainly included data regarding the name, gender, date of birth, date of death, cause of death, place of death, and other basic information of the participants. The community health service center and the Center for Disease Control and Prevention (CDC) collected the death certificates of residents from PSB’s household registration department on a monthly basis. They built a database of the death causes of residents in PNA (10). All causes of death were coded by strictly trained clinicians according to the actual situation of patients with predefined coding rules and further checked by the CDC to ensure the homogeneity and accuracy of the data (8).

### ICD-10 codes

International Classification of Diseases (ICD) is a system expressed by coding, which classifies diseases according to some characteristics of diseases and certain rules. At present, the mature version is ICD-10,^1^ which standardizes and formats the names of causes of death, and provides a research basis for analyzing the international comparability of mortality data (11). According to the ICD-10, acute myocardial infarction has code I21, including acute transmural myocardial infarction of the anterior wall (I21.0), acute transmural myocardial infarction of the inferior wall (I21.1), acute transmural myocardial infarction of other sites (I21.2), acute transmural myocardial infarction of unspecified site (I21.3), acute subendocardial myocardial infarction (I21.4), acute myocardial infarction, unspecified (I21.9). We use the recommended method to evaluate data quality which defines and classifies garbage codes into different types (10). In addition, the definition of “died with AMI” means all-cause of death included AMI. Meanwhile, “died of AMI” means the underlying cause of death was AMI.

### Statistical analyses

The primary outcome parameter of the study was mortality. Segi’s world population composition was used as the referenced population to standardize the mortality of AMI. Subsequently, CMR and ASMRW of AMI were calculated and presented as data per 100,000 persons (/10^5^). Z test and Mantel–Haenszel test were used to compare if CMR and ASMRW were different by gender of the study population. YLL refers to the number of years lost due to a certain disease, which can reflect the social burden of the disease more accurately (7). The efficacies of the interventions were analyzed by evaluating the changes in premature death and the mortality differences of different subgroups, as indicated by YYL (12). In this study, YLL was used to analyze the burden of AMI according to the method proposed by Murray and Lopez (13).

Ages were divided into eight groups according to previous studies (6). However, considering that there are no or very few deaths due to AMI in residents younger than 30 years old, the calculation and trend analysis of age-specific CMR, age-specific proportion, and age-specific YLLr are only performed in the following six age groups: 0–29 years, 30–44 years, 45–59 years, 60–69 years, 70–79 years, and ≥80 years. Joinpoint regression model has been widely used in disease mortality trend research (14). Therefore, we used Joinpoint regression to calculate the CMR, ASMRW, and YLLr over time and expressed the degree of change trend with average annual percent change (AAPC) and its 95% confidence interval (CI).

The Bayesian Information Criterion (BIC) was used to compare whether AAPC values were statistically different from zero. When the difference was statistically significant (*p* < 0.05), it indicated that the trend presented an “increase” or “decrease” change, and when the difference was not, it indicated that the trend was “stable.” The quantitative contribution of demographic and non-demographic factors to the change in AMI mortality was evaluated by the decomposition method (15). The statistical analyses were performed using SPSS (version 26.0; SPSS, Inc., Chicago, IL), R (version 3.4.3), and the Joinpoint Regression Program 4.3.1.0 (National Cancer Institute, Bethesda, MD, United States; source: https://surveillance.cancer.gov/joinpoint/), with *p* < 0.05 indicating a statistically significant difference.

## Results

### Baseline characteristics

**Figure 1:**
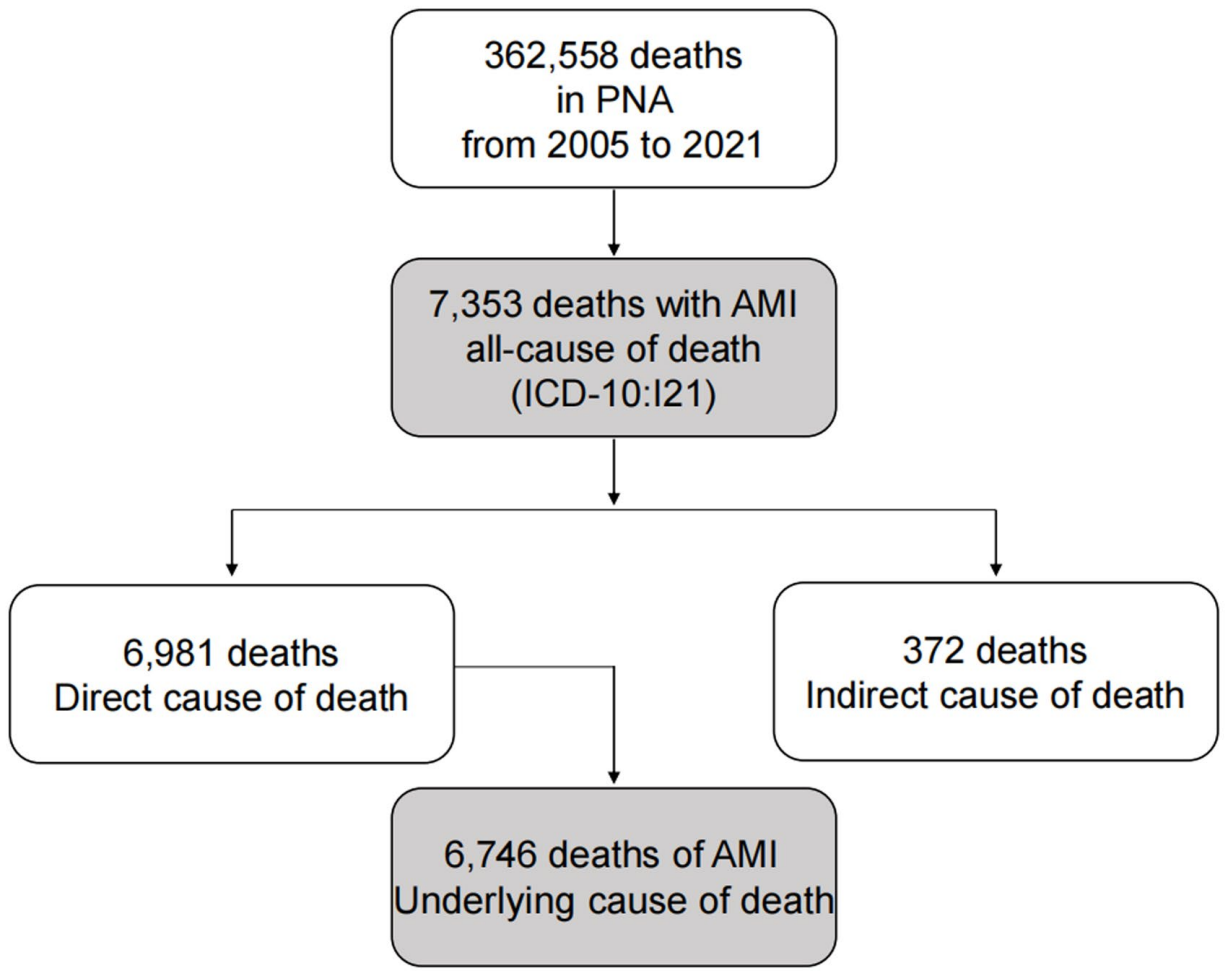
Flow chart for the selection of AMI-related deaths in PNA, Shanghai from 2005 to 2021.

From 1 January 2005 to 31 December 2021, a total of 48,276,547 registered residents in PNA were included in this study, and a total of 362,558 people died during the period. Specifically, 7,353 of the participants died with AMI, including 6,746 participants who died of AMI (Figure 1). For participants who died with AMI, the average age at death was 78.11 ± 11.15 years, and the median age at death was 80.27 years. The CMR and ASMRW were (15.23/10^5^and 5.17/10^5^) person-years, higher in males than in females (*U* = 10.01, *p* < 0.001; *Z* = −22.06, *p* < 0.001) and the proportion of females was 44.27%. The CMR, ASMRW, and the YLLs, YLLr of both gender are presented in Table 1. In addition, the YLLr of participants in 60–69 years, 70–79 years, and ≥ 80 years were 196.83/10^5^, 527.84/10^5^, and 937.59/10^5^, respectively (Table 2). For participants who died of AMI, the CMR and ASMRW were (13.97/10^5^and 4.73/10^5^) person-years, higher in males than in females (*U* = 9.42, *p* < 0.001; *Z* = −21.07, *p* < 0.001) and the proportion of females was 44.37% (Table 1).

**Table 1:** Baseline characteristics of the residents who died with/of AMI during 2005–2021.

Characteristic	Deaths (*n*, %)	Age at death (mean ± SD)	Age at death (median)	Age at death (range)	CMR (/10^5^)	ASMRW (/10^5^)	YLL (years)	YLL rate (/10^5^)
**In the residents death with AMI**								
**Gender**								
Male	4,098(55.73)	75.31 ± 11.83	77.47	20.19–101.65	17.01	6.87	37308.07	154.88
Female	3,255(44.27)	81.64 ± 9.09	83.06	22.47–106.29	13.46	3.61	24583.41	101.63
Total	7,353(100.00)	78.11 ± 11.15	80.27	20.19–106.29	15.23	5.17	61891.48	128.20
**The top 3 underlying cause of death of residents died with AMI**								
Coronary heart disease (I20–I25)	6,769(92.06)	78.2 ± 11.21	80.38	20.19–106.29	14.02	4.75	80231.26	166.19
Cerebrovascular disease (I60–I69)	141(1.92)	79.24 ± 9.43	81.88	50.12–94.00	0.29	0.09	1687.97	3.50
Diabetes mellitus (E10–E14)	139(1.89)	76.63 ± 9.45	77.95	42.48–97.03	0.29	0.12	1889.32	3.91
**In the residents death of AMI**								
**Gender**								
Male	3,753(55.63)	75.27 ± 11.92	77.52	20.19–101.65	15.58	6.30	34236.05	142.13
Female	2,993(44.37)	81.89 ± 8.96	83.29	22.47–106.29	12.37	3.30	22347.96	92.39
Total	6,746(100.00)	78.20 ± 11.20	80.38	20.19–106.29	13.97	4.73	56584.01	117.21
**The top 3 comorbid diseases of residents died of AMI (a person suffering from multiple diseases of the same ICD code is calculated only once)**								
Coronary heart disease (I20–I25)	6,746(34.24)	78.2 ± 11.20	80.38	20.19–106.29	13.97	4.73	79947.90	165.60
Hypertensive diseases (I10–I15)	3,013(15.29)	78.61 ± 10.17	80.21	27.23–106.29	6.24	2.08	35458.99	73.45
Heart disease (I05–09, I16–19, I26–I27, I30–I52)	1813(9.20)	77.81 ± 11.1	79.98	30.28–100.12	3.76	1.28	21621.96	44.79

CMR, crude mortality rate; ASMRW, age-standardized mortality rate by Segi’s world standard population; YLL, years of life lost.

**Table 2:** Age-specific mortality and burden of residents who dies with/of AMI during 2005–2021.

Age group (years)	Deaths (*N*)	Proportion (%)	CMR (/10^5^)	YLL (years)	YLL rate (/10^5^)
**Residents who died with AMI**					
0–29	8	0.11	0.11	216.24	2.87
30–44	68	0.92	0.62	1610.39	14.70
45–59	469	6.38	3.80	8689.97	70.39
60–69	965	13.12	14.22	13355.49	196.83
70–79	2064	28.07	57.60	18914.69	527.84
≥80	3,779	51.39	185.46	19104.68	937.59
Total	7,353	100.00	15.23	61891.48	128.20
**Residents who died of AMI**					
0–29	8	0.12	0.11	216.24	2.87
30–44	62	0.92	0.57	1464.71	13.37
45–59	437	6.48	3.54	8093.17	65.56
60–69	857	12.70	12.63	11865.39	174.87
70–79	1891	28.03	52.77	17336.89	483.81
≥80	3,491	51.75	171.33	17607.60	864.12
Total	6,746	100.00	13.97	56584.01	117.21

CMR, crude mortality rate; YLL, years of life lost.

### Main comorbidities

**Figure 2:**
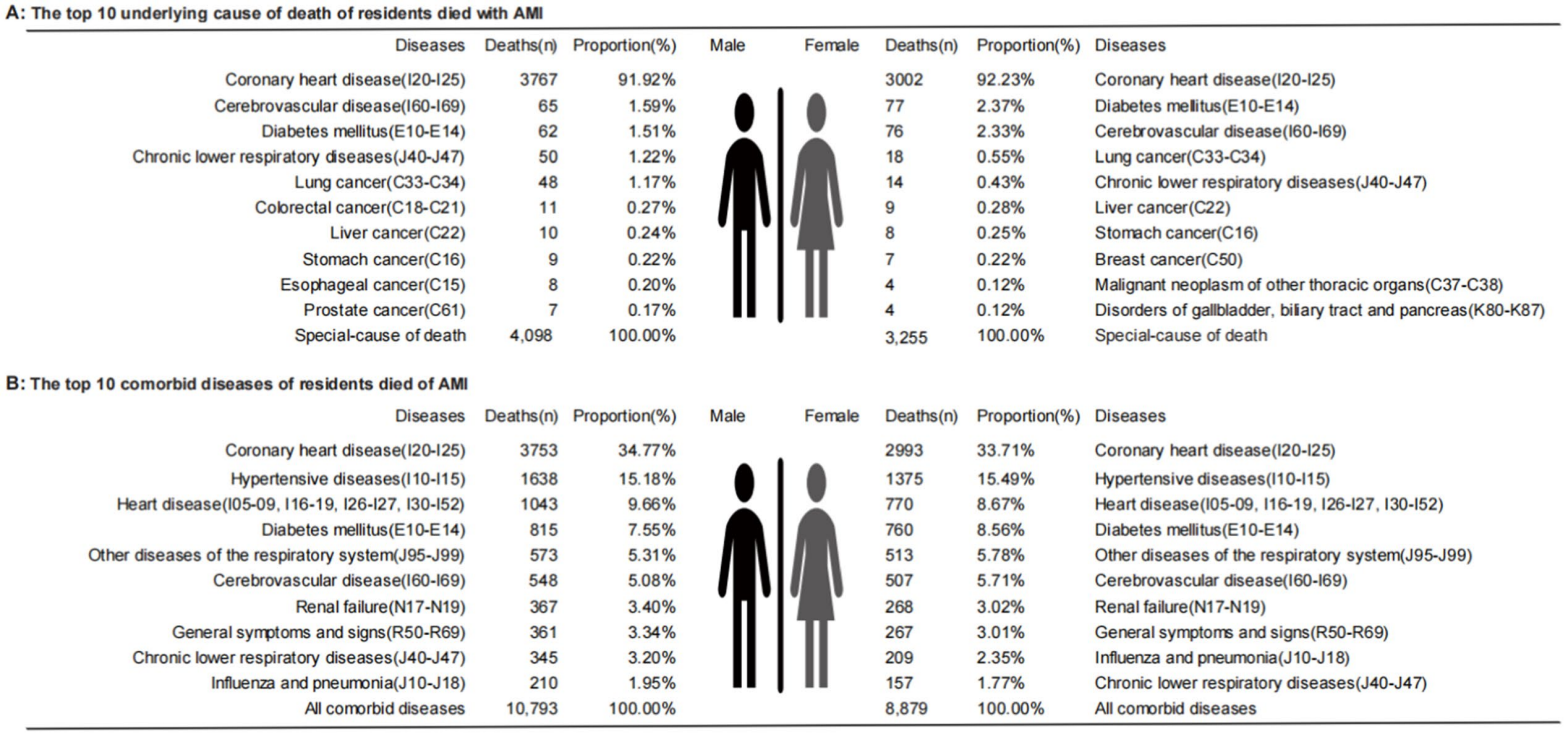
Sequence map of causes of death by gender. **(A)** The top 10 underlying cause of death of residents died with AMI; **(B)** The top 10 comorbid diseases of residents died of AMI.

For participants who died with AMI, the top 3 prevalent comorbidities were coronary heart disease (92.06%), cerebrovascular disease (1.92%), and diabetes mellitus (1.89%; Table 1; Supplementary Table S1). The top 10 comorbidities for participants who died with AMI by gender are shown in Figure 2A. For participants who died of AMI, the top 3 prevalent comorbidities were coronary heart disease (34.24%), hypertensive diseases (15.29%), and heart disease (9.20%; Table 1; Supplementary Table S2), and the gender-specific data are shown in Figure 2B. For participants who died with/of AMI, the spectrum types, rankings, and proportions of specific comorbidity can be found in Supplementary Tables S1, S2, and the data of YLLs, YLLr (Table 1), and the number of comorbidities (Supplementary Table S3) are also presented.

### Trends of burden

**Figure 3:**
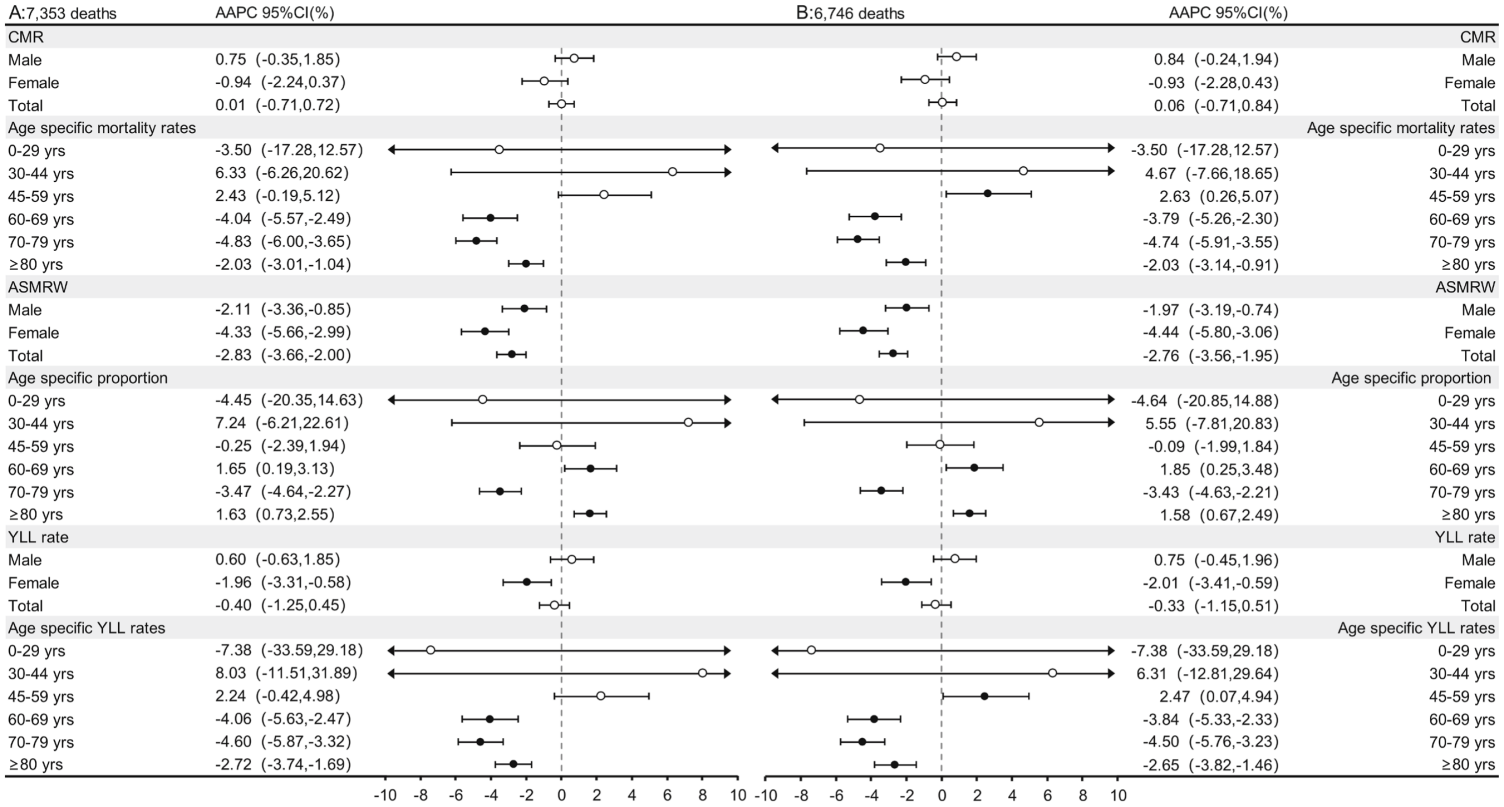
The trends in CMR, ASMRW, age specific proportions, and YLL of residents who died with/of AMI in genders and age groups in PNA, Shanghai, China, 2005–2021. **(A)** The trends in CMR, ASMRW, age specific proportions, and YLL of residents who died with AMI; **(B)** The trends in CMR, ASMRW, age specific proportions, and YLL of residents who died of AMI. CMR, crude mortality rate (per 100,000); ASMRW, age-standardized mortality rate by Segi’s world standard population (per 100,000); YLL, year of lost. AAPC, average annual percent change; CI, confidence interval.

The AAPC value of CMR of residents who died with AMI was 0.01% (95% CI: −0.71,0.72, *p* = 0.989), in the overall participants but increased in males and decreased in females (all *p* > 0.05). However, the ASMRW of both gender showed a downward trend, which was −2.83% (95% CI: −3.66,−2.00, *p* < 0.001; Figure 3A). Similar trends were also observed in deaths of AMI (Figure 3B). The case number, proportion, and CMR of the residents who died with/of AMI in different age groups are shown in Table 2. The case number, CMR and ASMRW of the residents who died with/of AMI according to gender in each year are shown in Supplementary Tables S4, S5 and the CMR data in different age groups of each year are shown in Supplementary Table S6. The trends of the proportion of the different types of deaths in PNA are shown in Supplementary Figure S1, and the population aging trend chart of PNA, Shanghai, is shown in Supplementary Figure S2. The YLLr by gender and age groups and the proportion by age groups of death with/of AMI in PNA are shown in Supplementary Tables S7, S8. The median age at death of residents died of AMI in PNA is shown in Supplementary Table S9.

For deaths with AMI, the total YLLr decreased by 0.40% (95% CI: −1.25,0.45, *p* = 0.331) per year. A gender difference was observed, which was increased in males (*p* = 0.318) but decreased in females (*p* = 0.009). Analyses according to the age group showed that for participants of ≥60 years, a downward trend was observed, and the age-specific YLLr increased by −4.06% (95% CI: −5.63 to −2.47%), −4.60% (95% CI: −5.87 to −3.32%), and − 2.72% (95% CI: −3.74 to −1.69%) per year, all *p* < 0.001, respectively (Figure 3A). Similar results were also observed for participants who died of AMI (Figure 3B). Interestingly, an age-group difference was found between residents who died of and died with AMI. Specifically, among the middle-aged (45–59 years) group, the YLLr increased by 2.47% (95% CI: 0.07,4.94, *p* = 0.045) per year, which showed an upward trend in those who died of AMI. The YLLs and YLLr of the deaths with/of AMI by age groups are shown in Table 2.

### Quantitative analyses for the influences of the demographic and non-demographic factors on mortality

**Figure 4:**
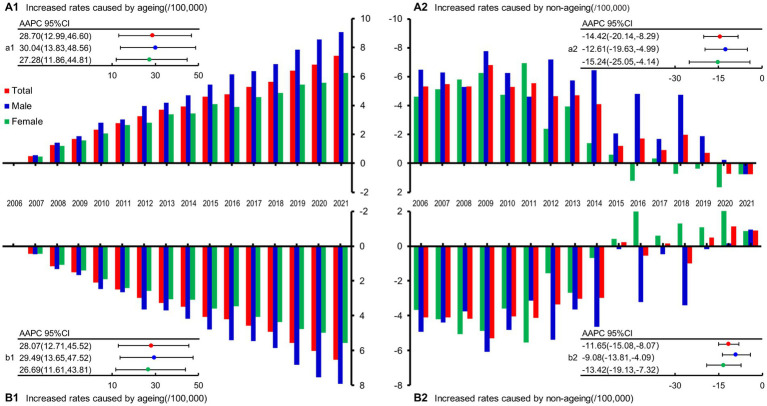
Influences of demographic and non-demographic factors on AMI-related deaths during the period from 2005 to 2021 in PNA, Shanghai, China. **(A1)** The increased rates caused by demographic age structure in death with AMI; **(A2)** The increased rates caused by non-demographic age structure in death with AMI; **(a1)** The trend of the mortality rate caused by demographic age structure in death with AMI; **(a2)** The trend of the mortality rate caused by non-demographic age structure in death with AMI; **(B1)** The increased rates caused by demographic age structure in death of AMI; **(B2)** The increased rates caused by non-demographic age structure in death of AMI; **(b1)** The trend of the mortality rate caused by demographic age structure in death of AMI; **(b2)** The trend of the mortality rate caused by non-demographic age structure in death of AMI; AAPC, average annual percent change; CI, confidence interval.

Among deaths with AMI, the total effects of demographic and non-demographic factors on CMR were different, with AAPC of 28.70% (95% CI: 12.99,46.60, *p* = 0.001) and −14.42% (95% CI: −20.14,−8.29, *p* < 0.001), respectively. In males, the AAPC for the effects of demographic and non-demographic factors were 30.04% (95% CI: 13.83,48.56, *p* = 0.001) and − 12.61% (95% CI: −19.63,−4.99, *p* = 0.004) respectively. For females, the AAPC for the effects of demographic and non-demographic factors were 27.28% (95% CI: 11.86,44.81, *p* = 0.001) and −15.24% (95% CI: −25.05,−4.14, *p* = 0.012; Figures 4A1,2). In addition, compared with participants who died with AMI, the influences of demographic and non-demographic factors on CMR in deaths of AMI were similar but weaker (Figures 4B1,2). The increments of CMR caused by demographic and non-demographic factors between 2005 and 2021 are also displayed in Supplementary Table S10.

## Discussion

Over the past 40 years of reform and opening up, China’s economy has developed rapidly. Although people’s physical living standards have greatly improved, the gross domestic product (GDP) *per capita* level still has room for improvement. However, chronic diseases are also prevalent among Chinese residents, particularly for CVDs (16). According to the calculation, the current number of patients with CVDs in China is 330 million, including 11.39 million patients with coronary heart disease (CHD) (16). As the most common acute and critical disease in CHD (3), AMI is associated with high morbidity and mortality and is an economic burden for the Chinese population (17). As a typical transition region in China, research regarding the trend of changes in the death characteristics and burden of AMI in PNA may provide information on the prevalence of AMI-related deaths in similar regions in the transition process, which may be critical for the subsequent development of effective preventative strategies.

### Burden of AMI

From Figure 3, the total CMR of AMI in PNA showed a slight upward trend from 2005 to 2021, although the difference was not statistically significant. Interestingly, the ASMRW after age structure adjustment diminished this trend. Analysis of the total population showed a significant downward trend, which was more remarkable for the females. At the same time, combined with the aging trend chart in PNA (Supplementary Figure S2), it became obvious that aging factors play a key role in the increase of CMR. This is consistent with earlier findings of a study by Chang et al. (18), which showed that the increase in AMI mortality was primarily attributable to population aging.

In terms of age groups, the CMR of the groups of the older adults (60–69 years, 70–79 years, and ≥80 years) all showed downward trends. This trend was similar to the conclusion of a previous German study, which showed a decline in AMI-related mortality in the older adult population (>64 years), indicating progress in prevention and treatment for the older adult population (19). Similar to Germany (developed country), China (developing country) can also achieve such prevention and control effects of AMI. The reason is inseparable from the implementation of the National Basic Public Health Service Project in China (20). The project (21) stipulates that health records should be established for the older adults aged 60 and above, and health care services and management should be executed in accordance with technical specifications and requirements, including providing community day care services and home-based door-to-door services such as living and medical care. In addition, based on the establishment of health records, health management services for the older adults aged 65 and above are provided once a year, including lifestyle and health status assessment, Traditional Chinese Medicine (TCM) constitution identification, physical examination, auxiliary examination, and health guidance. Similar services for newborns and pregnant women have achieved remarkable benefits in reducing neonatal and maternal mortality (22, 23). This project can also be expected to serve as a positive example for other developing countries looking to reduce AMI-related mortality in the older adults.

However, unexpected findings of increased AMI-related deaths were observed in the middle-aged population. In the 45–59 years age group (Figure 3), the CMR and YLLr showed an increasing trend by year, which to our knowledge, has not been reported in similar studies (18, 19, 24). People in this age group are in the transition period from youth to old. They are “middle-aged” adults, but they have a trend of increased mortality and higher YLL (Table 2). An analysis of 1,462,168 young adults with their first AMI showed that, in participants aged 45–59 years, hypertension (59.8%), dyslipidemia (57.5%), and smoking (51.9%) were most prevalent, and 92% of patients had at least one risk factor (25). These findings suggest that the prevention of AMI-related death in this “middle-aged” population may be the key objective in the future. For example, the age limits of the service of the national project mentioned above should be younger if permitted, e.g., from above 60 years to over 45 years old or 50 years old. Further cost-effective studies should also be performed to validate this strategy. For example, a previous study in India confirms that policies to expand treatment and preventive care for AMI are cost-effective based on GDP *per capita* comparisons (26). Although we have seen a downward trend in the CMR of the older adults and the proportion of AMI in all death (Supplementary Figure S1), the rising trend in the proportion of AMI deaths in the last few years may be related to the lack of effective treatment due to stress, closed management, and medical resource runs caused by COVID-19 (27). We should also see that there are differences in this trend among different age groups since the proportion of deaths with/of AMI in the number of deaths in this age group still has a certain upward trend in some age groups (Figure 3). For participants aged 60–69 years and ≥80 years (Figure 3 Age specific proportion), measures for the older adults in the above-mentioned national project should continue to be implemented and strengthened.

### Main comorbidities

Comorbidities have been related to the poor long-term survival of patients after AMI (28). Pathophysiologically, AMI is mainly caused by coronary atherosclerotic stenosis in patients with CHD (29). During the pathogenesis of AMI, the coronary atherosclerotic plaque ruptures, and the platelets in the blood gather on the surface of the ruptured plaque, forming blood clots (thrombi), which suddenly block the coronary artery lumen, resulting in acute myocardial ischemic necrosis (3). Therefore, from Table 1, it can be seen that CHD is the first comorbid disease of AMI, whether it is in participants who died with or of AMI. At the same time, we also found that cerebrovascular disease and diabetes mellitus also account for a higher proportion of dying with AMI. Hall et al. (28) also found that the coexistence of multiple diseases in patients with AMI is very common, primarily with diseases such as hypertension, heart failure, and peripheral vascular disease. Our findings support that most residents who died of AMI also have multiple cardiovascular diseases such as hypertension and heart diseases.

In addition, from the gender-specific analyses in Figure 2, besides CVDs, diabetes is within the top 3 comorbidities for both genders. Diabetes can lead to several serious complications in the heart, blood vessels, kidneys, eyes, and nerves (30). Diabetes has a high prevalence in the Chinese population. A survey from 2015 to 2017 showed that the prevalence of diabetes in Chinese people aged ≥ 18 was 11.2%, which has contributed significantly to the burden of mortality and morbidity (30, 31). Globally, the number of people with diabetes quadrupled in more than 30 years between 1980 and 2014, reducing the life expectancy and causing severe loss of life (32), which has a strong public health impact worldwide (33). In the past 10 years, the prevalence of diabetes with AMI also increased (34). Our study showed that diabetes is within the top 4 in death with/of AMI (Supplementary Tables S1, S2) and causes a severe loss of life in PNA residents (Table 1). Although genetic factors may have a certain influence on the high prevalence of diabetes, it is generally believed that diabetes is related to unhealthy lifestyles (35), and a healthy lifestyle also has a certain counteracting effect on genetic susceptibility (36). Therefore, health guidance is necessary for the high-risk population of diabetes, too.

Besides, chronic obstructive pulmonary disease and lung cancer may also have great impacts on early death with AMI. Although there is little difference in the ranks of respiratory comorbidities in men and women, they all rank within the top 5 of comorbidities (Figure 2A). The cardiovascular and pulmonary systems often interact with and influence each other, and injury to one system will also affect the other, leading to the occurrence of diseases (37–39). Therefore, in the secondary prevention of CHD progressing to AMI, treatments for respiratory comorbidity should be regarded as the concomitant treatment goal of improving cardiovascular outcomes.

### Demographic and non-demographic factors

Among demographic factors, the impact of population aging on mortality is very important. The expert panel of the “China Cardiovascular Health and Disease Report 2021” (16) pointed out that China is facing the pressure of an aging population, and the CVD burden will continue to increase. Research shows that PNA took 26 years from being an aging society (in 1982) to an aged society (in 2008), while it took only 10 years from being an aged society to a super-aged society (in 2018) (7). Our research (Figure 4; Supplementary Figure S2) also confirmed that population aging in PNA is accelerating. Analyzed by gender, the aging of both men and women who died with/of AMI both showed an upward trend, but the trend in men was slightly higher than that of women (Figure 4), which may be related to the lower baseline life expectancy of men (40). Non-demographic factors mainly included economic development, the level of medical technology, the accessibility of medical services, health literacy of the residents, environmental factors, etc. (7). Figure 4 showed that non-demographic factors have hindered the increase in the CMR of AMI, indicating that the corresponding public health policies and specific clinical intervention measures have played positive roles in the prevention of AMI-related deaths (41).

With the increase in the contribution of aging to the CMR, the proportions of non-demographic factors have also gradually decreased (Figure 4). In gender-specific analyses, although a downward trend was shown in both men and women, the decline is faster for women (Figures 4A2,B2), indicating that the impact of intervention measures on women can be strengthened. A British study also confirmed that according to AMI quality indicators, women less frequently received guideline-indicated care and had significantly higher severity than men (42) and the use of measures for secondary prevention treatment is particularly low in women (43). The female population should be listed as the key target population in this regard, and women should receive greater attention regarding the delivery of recommended AMI prevention and treatment measures.

Further analyses of the overall population showed that the contribution of aging factors to mortality is greater than that of non-demographic factors in death with/of AMI. With the development of the economy and the progress of medical care, the average life expectancy has correspondingly prolonged, and aging has become an irreversible trend in various countries. Therefore, how to age healthily has become an important topic in the field of modern medical research (44). At present, the life expectancy of Shanghai residents in 2021 is 84.11 years old, which is 81.76 years for men and 86.56 years for women (http://wsjkw.sh.gov.cn/tjsj2/20220704/a540b90305ae4c54bf870b3804c6f84c.html, first line of the first paragraph). The median age of people who die of AMI is 81.16 years in 2021, which is close to the above life expectancy. This may be related to the “1 + 1 + 1” signing service (http://www.gov.cn/xinwen/2016-12/27/content_5153507.htm, in paragraph 2), which has become an important measure to implement healthy aging, implemented in Shanghai (45, 46). Specifically, the “1 + 1 + 1” signing service includes a general practitioner, a district-level medical institution, and a municipal-level medical institution, giving priority to meeting the signing needs of the older adult over 60 years old with chronic diseases. After signing the contract, residents can enjoy convenient medical diagnosis and treatment, health care, health education, rehabilitation, and other services. By providing continuous, comprehensive, and life-cycle signing services, every older adult is expected to have their own family doctor who knows their condition and who will help them achieve healthy aging (47).

In addition, the role of environmental problems in human health has also attracted more attention from scientists. Studies have shown that transient exposure to air pollutants, including PM (2.5), NO_2_, SO_2_, etc., may be important inducing factors for AMI, even at concentrations below the World Health Organization air quality guidelines (48, 49). Besides solving the traditional cardiovascular disease risk factors, the government must also formulate relevant policies to reduce the harm of air pollution to citizens’ health (49). In recent years, China has issued a series of policies and regulations to control air pollution in the form of legislation, including the first time in 2012 that China incorporated PM2.5 into the National Ambient Air Quality Standards (50). Economic development cannot be at the expense of the environment because clear waters and green mountains are as valuable as mountains of gold and silver. Whether it is the “1 + 1 + 1″ signing service or environmental legislation in non-demographic factors, we hope that healthy aging can be implemented and transformed from treatment-centered to health-centered.

### Future

China is facing the continuous pressure of the aging population and the control of metabolic risk factors, which has put forward new demands for CVD prevention and control strategies. These include optimization of the medical resource allocation, reducing the number of patients through primary prevention, increasing the allocation of medical resources for cardiovascular emergency treatment, and providing medical services of rehabilitation and secondary prevention to reduce the risk of recurrence, rehospitalization, and disability of a large number of CVD survivors. Specific prevention and treatment technologies should be implemented in different countries and regions to address the growing health needs associated with population aging, especially for patients with serious life-threatening diseases, such as AMI.

### Strengths and limitations

The strengths of this study include the followings. Firstly, we selected PNA as the region of study, which involves a population of more than 3 million and is a highly representative transitioning area. Therefore, the results of the study could provide important reference significance for the prevention and treatment of AMI in other regions or countries with similar economic development and aging trends. Secondly, we not only analyzed the CMR trend of the all-cause of death (dying with AMI) but also further analyzed the changing trend of AMI as the underlying cause of death (dying of AMI), as well as ASMRW and YLLr, which could provide a comprehensive dataset. Besides, we also quantitatively analyzed the effect of demographic and non-demographic factors on CMR, which to our knowledge, has not been reported in other similar literature. Finally, AMI, as an acute and critical disease, differs from other chronic diseases. AMI often has typical symptoms, an evolution process, and strict diagnostic criteria, and researches regarding all-cause or underlying cause deaths related to AMI are likely to be more reliable. Despite these strengths, this study also has limitations. For example, due to the limitation of conditions, this study only includes the death data of residents in PNA for 16 years, not like the GBD 2019 for 30 years, and the impact of the intervention measures taken in this area on the changes of AMI mortality needs to be further observed. In addition, this study only discussed the impact of aging factors on the long-term changes of the CMR in AMI but did not discriminate against non-aging factors. At last, this study is an observational study, and the causal relationship between influencing factors and mortality changes in AMI could not be derived based on the findings.

## Conclusion

Aging population and comorbidities may have played a role in the difference in trends between CMR stability and ASMRW decline in AMI in the transitioning region. Preventative strategies should be applied to enhance the health management of residents aged 45–59 years or with comorbidities, such as improving the lifestyle, targeted management of comorbidities, and intensified medical treatments for AMI.

## Data availability statement

The raw data supporting the conclusions of this article will be made available by the authors, without undue reservation.

## Ethics statement

This study was approved by the Ethics Committee of the School of Public Health, Fudan University (No: IRB#2016-04-0586). Written informed consent for participation was not required for this study in accordance with the national legislation and the institutional requirements.

## Author contributions

YZ and XL: conceptualization, methodology, software, and writing—original draft. YZ, JZo, and YC: formal analysis. XL and SJ: validation and funding acquisition. JZo, YC, JZh, WD, and MP: writing—review and editing. All authors contributed to the article and approved the submitted version.

## Funding

This study was funded by a grant from Shanghai Municipal Program for Medical Leading Talents (2019LJ15 to SJ) and Shanghai Public Health System Construction Three-year Action Plan Outstanding Youth Talent Training Program (GWV-10.2-YQ43 to YC).

## Conflict of interest

The authors declare that the research was conducted in the absence of any commercial or financial relationships that could be construed as a potential conflict of interest.

## Publisher’s note

All claims expressed in this article are solely those of the authors and do not necessarily represent those of their affiliated organizations, or those of the publisher, the editors and the reviewers. Any product that may be evaluated in this article, or claim that may be made by its manufacturer, is not guaranteed or endorsed by the publisher.

## Footnotes

^1^https://www.cdc.gov/nchs/icd/icd10.htm

## Abbreviations

AAPC: Average annual percent change
AMI: Acute myocardial infarction
APC: Annual percentage change
ASMRW: Age-standardized mortality rates worldwide
CDC: Center for Disease Control and Prevention
CHD: Coronary heart disease
CI: Confidence interval
CMR: Crude mortality rates
CVD: Cardiovascular disease
GBD: Global Burden of Disease
GDP: Gross domestic product
ICD: International Classification of Diseases
PNA: Pudong New Area
PSB: Public Security Bureau
RMB: Ren Min Bi
TCM: Traditional Chinese Medicine
YLLr: rates of Years of life lost
