# Supplementary figures and images for: Changes of the acute myocardial infarction-related resident deaths in a transitioning region: a real-world study involving 3.17 million people
Source: Front Public Health. 2023 Aug 21;11:1096348. doi: 10.3389/fpubh.2023.1096348 (PMC10476525; doi:10.3389/fpubh.2023.1096348)

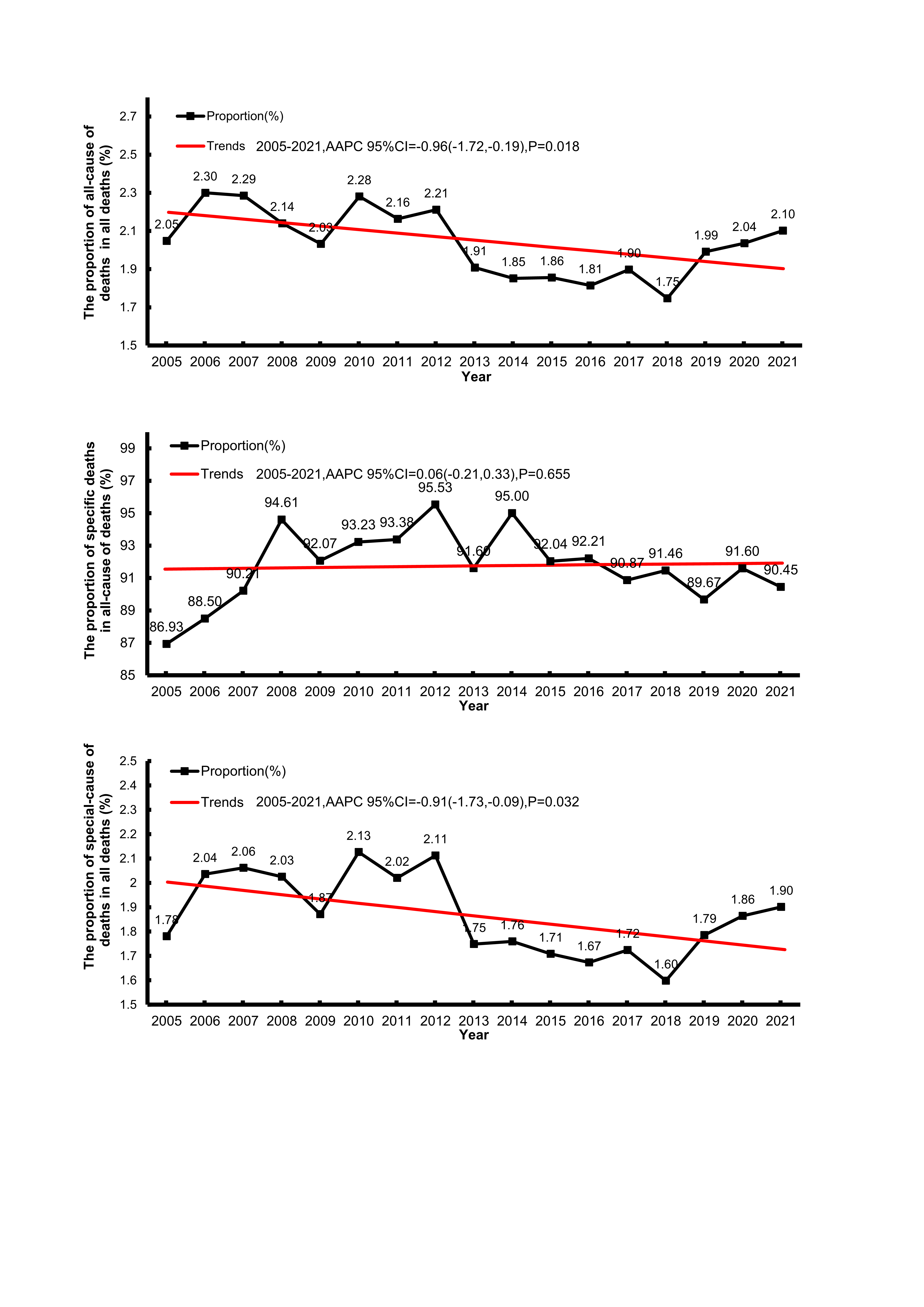

Supplement: Supplementary file 1 [file Data_Sheet_1.zip › FigS1.tif]

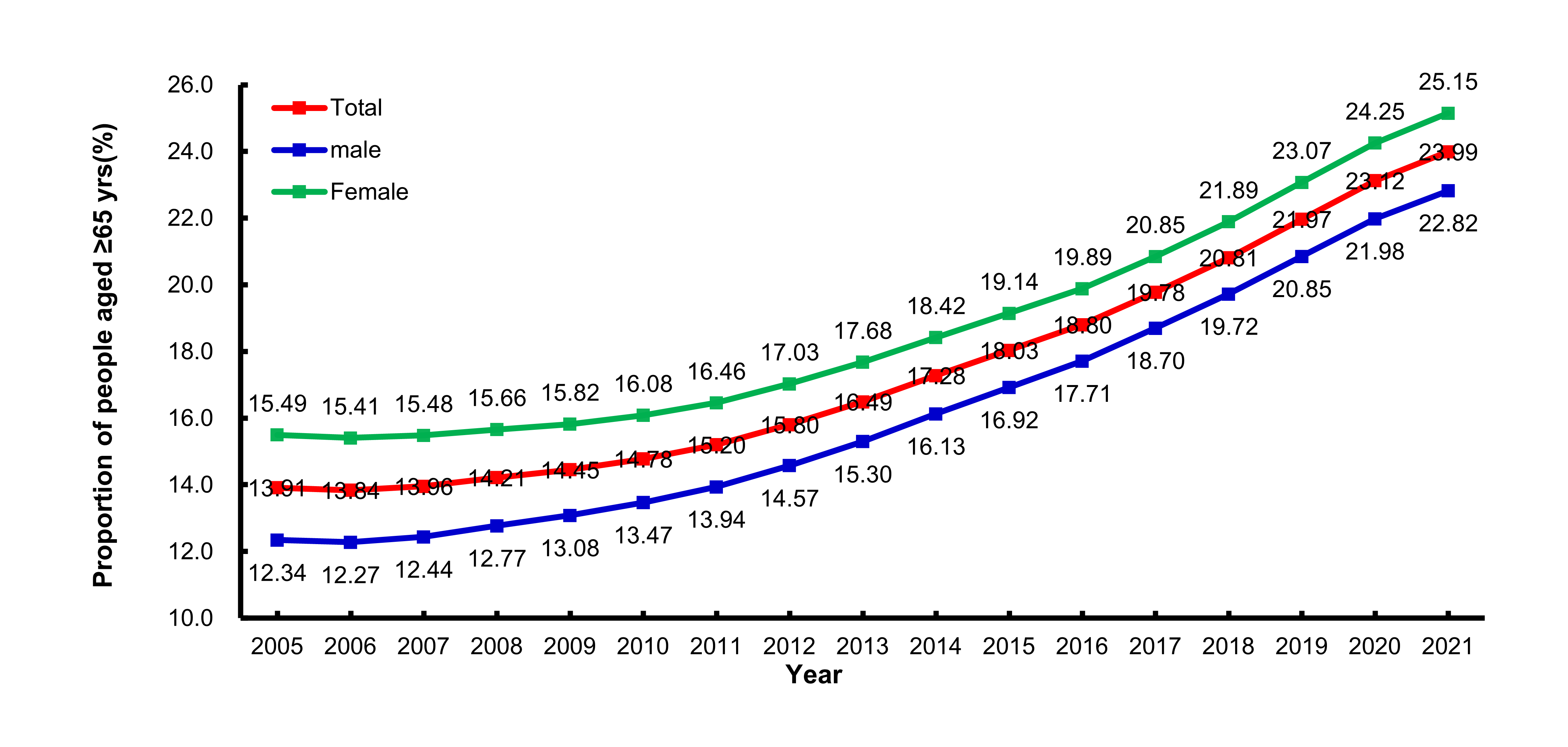

Supplement: Supplementary file 1 [file Data_Sheet_1.zip › FigS2.tif]
